# Comparison of Therapeutic Outcomes of Transabdominal Pararectus Approach and Modified Stoppa Approach in Treating Pelvic and Acetabular Fractures

**DOI:** 10.1007/s43465-021-00585-1

**Published:** 2022-01-03

**Authors:** Wei Liu, Hongbin Yang, Zhenyan Yu, Yu Zhao, Jigong Hu, Benyang Li, Yechong Zhu

**Affiliations:** 1 Department of Orthopedics, Lu’an Hospital of Chinese Medicine, Lu’an, 237006 Anhui Province China; 2 Department of Orthopedics, Lu’an Hospital Affiliated to Anhui University of Chinese Medicine, Lu’an, 237006 Anhui Province China; 3 School of Pharmacy, Bengbu Medical College, Bengbu, 233030 Anhui Province China

**Keywords:** Pelvis, Acetabulum, Pararectus abdominis approach, Stoppa approach, Fracture fixation

## Abstract

**Objective:**

Pelvic and acetabular fractures are common orthopedic diseases, and this research was to investigate the therapeutic effects of pararectus and Stoppa approaches in treating complex pelvic acetabular fractures.

**Methods:**

The clinical information of patients with pelvic and acetabular fractures treated surgically in Lu'an Hospital of Chinese medicine, China from January 2016 to April 2020 was analyzed. There were 30 cases each in the transabdominal pararectus approach and modified Stoppa approach groups. The operation time, incision length, blood loss, and postoperative complications of both groups were recorded according to the Merle d'Aubigné-Postel hip score. The recovery of hip function was evaluated 6 months after surgery, and the clinical and therapeutic efficacies of the two groups were compared.

**Results:**

The patients were followed up for 6–7 months (average, 6.5 months). The average operation time, incision length, and blood loss in the pararectus and Stoppa approach groups were 180 ± 41.105 min, 8.667 ± 1.373 cm, 259.667 ± 382 mL and 202.667 ± 32.793 min, 11.600 ± 1.958 cm, and 353.667 ± 590 mL, respectively. The satisfactory rate of fracture reduction, excellent and good rate of hip function score, and incidence of complications were 28/30, 27/30, 1/30 and 25/30, 25/30, 3/30, respectively. There were significant differences in operation time, incision length, and blood loss between the two groups (*p* < 0.05). However, there was no significant difference in the excellent and good rate of hip function score, fracture reduction satisfaction, and complication rate between both groups (*p* > 0.05).

**Conclusions:**

The pararectus approach can reveal the better anatomical structure of the pelvis and acetabulum, such as the corona mortis and quadrilateral plate, for conducive fracture reduction and fixation. It can also effectively shorten the length of the incision, reduce operative blood loss, and shorten the operation time. It is a better choice for the clinical treatment of complex pelvic and acetabular fractures.

## Introduction

Pelvic and acetabular fractures are becoming more common, generally because of severe high-energy trauma. Because of the complex anatomy of the pelvis and acetabulum and the complicated injury mechanisms, there is a consensus that surgical treatment of unstable pelvis and acetabulum fractures is one of the optimal choices if the patients are without surgical contraindications [1]. The inguinal approach is a classic anterior surgical approach in the treatment of pelvic and acetabular fractures, which is generally suitable for superior pubic rami fractures, anterior column or anterior wall fractures [2, 3]. However, it requires passing through the inguinal canal, thereby causing some degree of damage to the inguinal area and may significantly increase the risk of inguinal hernia [4]. Second, this method requires separation and pulling of blood vessels, lateral femoral nerve, and lymphatic vessels during the operation, which can increase the probability of injury of these structures and cause lymphedema, lymphatic fistula, infection, nerve paralysis, muscle dysfunction, and other postoperative complications [5]. Thirdly, the surgical field of vision via the ilioinguinal approach is easily compromised by the inguinal ligament and abdominal muscle, leading to non-exposure of the high iliac bone area and quadrilateral plate, thereby increasing the difficulty of fracture reduction [6].

In view of the limitations and shortcomings of the traditional inguinal approach, pararectus and the modified Stoppa approaches are two new surgical methods used clinically for the treatments of acetabular fractures in recent years. In 2007, Hirvensalo et al. improved the Stoppa approach for the treatment of pelvic and acetabular fractures, which has the advantages of easier exposure of operative field and less trauma [7]. However, this method cannot expose the ala of ilium. For those patients with sacroiliac joint dislocation, high anterior column fractures of the acetabulum with ala of ilium, as well as sacral fractures, the iliac fossa approach should be supplemented with the Stoppa approach to complete the operation. Furthermore, in some patients, the rectus abdominis need to be cut off and this may easily induce muscle injury. In 2012, Keel et al. successfully applied the pararectus approach to treat acetabular fractures for the first time [8]. In 2014, Farouk et al. used the lateral incision approach of the rectus abdominis to treat acetabular fractures and achieved satisfactory results [9]. Besides, the lateral rectus abdominis incision approach has been successfully used for the treatment of acetabular fractures in the anterior column and quadrilateral plate, which proves that this approach is safe and feasible [10]. The best indication for this approach is comminuted fracture of the anterior acetabulum involving the quadrilateral plate area combined with ipsilateral pelvic fracture.

In general, the advantages of the transabdominal pararectus approach and modified Stoppa approach can be listed as follows: (1) small incision, easy anatomic approach, shorter operation time, and less blood loss; (2) the separation direction of the deep fascia tissue is the same as that of the nerve and blood vessel, and the damage to nerve and blood vessels is minimal; (3) the lateral femoral cutaneous nerve is not injured; (4) the fracture site, especially the quadrilateral plate, can be exposed thoroughly and operated and fixed under direct vision; (5) the pelvic ring is exposed from the inner side of the pelvic ring, and plates and screws are inserted; (6) the incision does not pass through the inguinal area, thus avoiding injury to the inguinal ligament. However, to compare between transabdominal pararectus approach and the modified Stoppa approach, there are still controversies regarding which of these two methods is the better choice in the curative outcomes and safety of pelvic and acetabular fractures treatments [11, 12]. Therefore, herein, we retrospectively analyzed the clinical information of patients with pelvic and acetabular fractures treated in Lu'an Hospital of Chinese medicine, China from January 2016 to April 2020, to explore the differences in curative outcomes of the pararectus and the modified Stoppa approaches.

## Materials and Methods

### Study Population and Definitions

Case inclusion criteria were as follows and all of the three standards must be met: (1) the diagnosis of pelvic acetabular fracture was confirmed by radiography and computed tomography (CT) three-dimensional reconstruction, (2) patients were aged 18–60 years old, (3) according with surgical indications: displacement of acetabular roof fracture was more than 2 mm and fracture with medial, anterior, and posterior roof-arc angles was less than 45, 25, or 70° involve the weight-bearing dome, respectively; or the displacement of fracture in other parts of pelvis and acetabulum was more than 5 mm; or hip dislocation reduction failure.

Case exclusion criteria were as follows and those according with one of them were excluded: (1) severe osteoporosis, (2) the area of posterior wall fracture accounted for more than 40% of the total area of posterior wall of acetabulum, (3) patients with severe underlying diseases patients such as cancer or severe cardiovascular disease, et al., (4) patients with large fracture communication in the fracture of pelvis and acetabulum or contaminated open wounds in the abdomen, (5) combined with other surgical approaches, such as posterior-wall acetabular fractures fixation using Kocher-Langenbeck approach, (6) patients who were lost in follow-up.

According to the inclusion and exclusion criteria, we collected the data from patient files and follow up treated in the department of orthopedics of Lu'an Hospital of Chinese medicine, China from January 2016 to April 2020. And then they were divided into transabdominal pararectus approach group, modified Stoppa approach group, and other group according to the operation method. Finally, 30 cases were randomly selected from the first two groups respectively and a total of 60 cases were retrospectively analyzed. The basic information of the selected cases is shown in Table 1. There were no significant differences in age, sex, fracture classification, injury causes, or other general information between the two groups (*p* > 0.05).

**Table 1:** Comparison of preoperative general information between pararectus approach and Stoppa approach

	Pararectus approach group	Stoppa approach group	Statistical test quantity	*P* value
Gender (male/female)	20/10	19/11	x^2^ = 0.073	0.787
Age (years)	41.993 ± 10.972	41.400 ± 9.544	*t* = 1.395	0.163
Acetabular fracture	18	17		
Letournel classification: transverse/anterior column/T-type fracture	7/6/5	7/5/5	x^2^ = 0.062	0.969
Pelvic fracture	12	13		
Tile classification: B1/B2/B3/C1	3/3/4/2	2/4/4/3	x^2^ = 0.735	1.000
Cause of injury			x^2^ = 0.275	0.872
Crush injury	5	6		
Traffic accident injury	17	15		
Fall injury	8	9		

### Surgical Technique and Postoperative Care

**Fig. 1:**
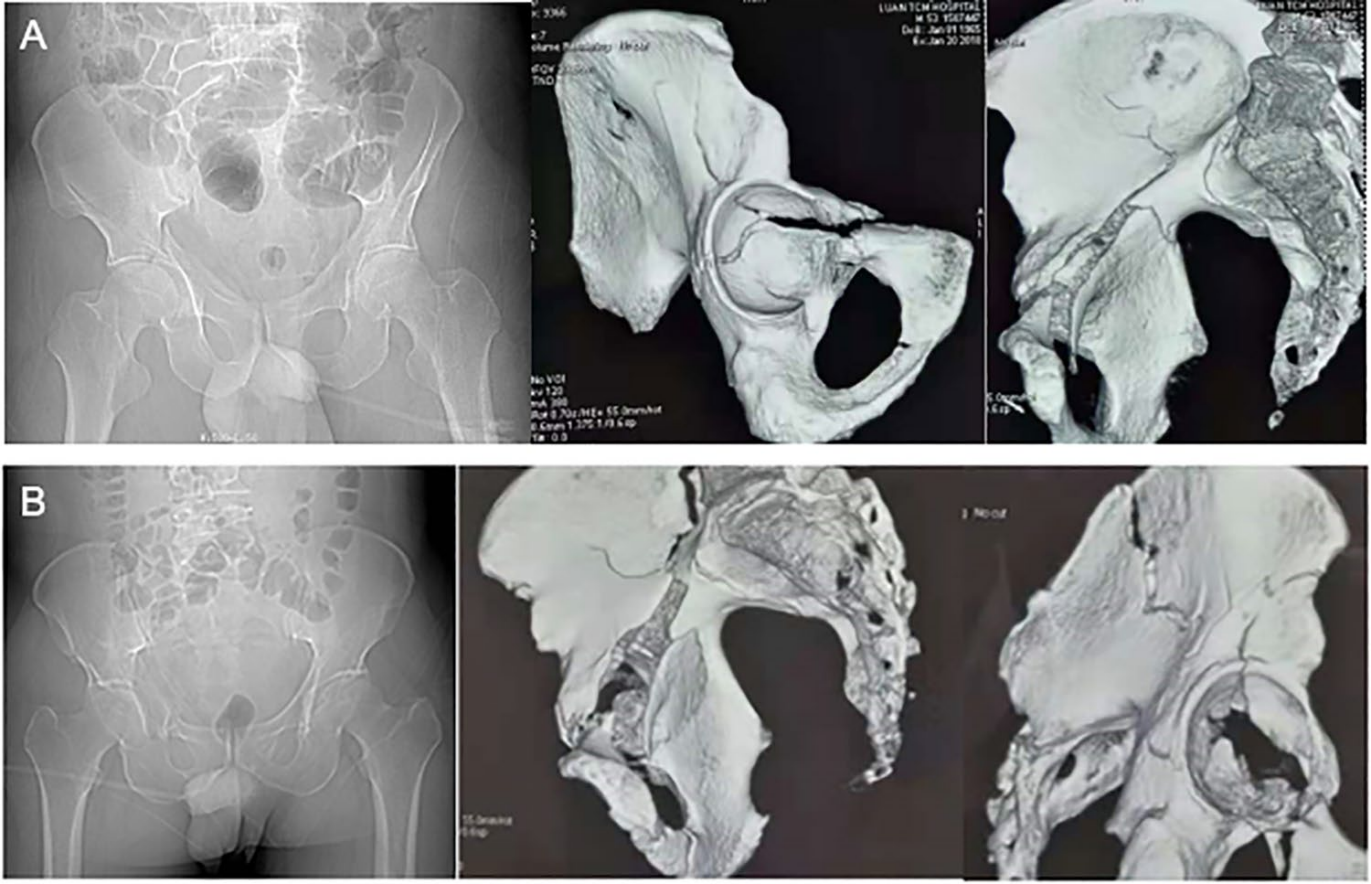
Preoperative X-ray and three-dimensional CT of the typical patient in **A** Stoppa approach group and **B** pararectus approach group

All patients were examined by digit radiography (DR) and three-dimensional CT (Fig. 1) before surgery to comprehensively evaluate the fracture range, classification, and morphology to determine the operation plan. All patients were treated with supracondylar femoral traction and tibial tuberosity bone traction before the operation. The surgery was scheduled 3–7 days after admission. General anesthesia was administered. The patients were placed in the supine position. The hip on the affected side was slightly raised. Surgical areas were routinely dissected. The two groups of patients were operated on by the same group of doctors.

**Fig. 2:**
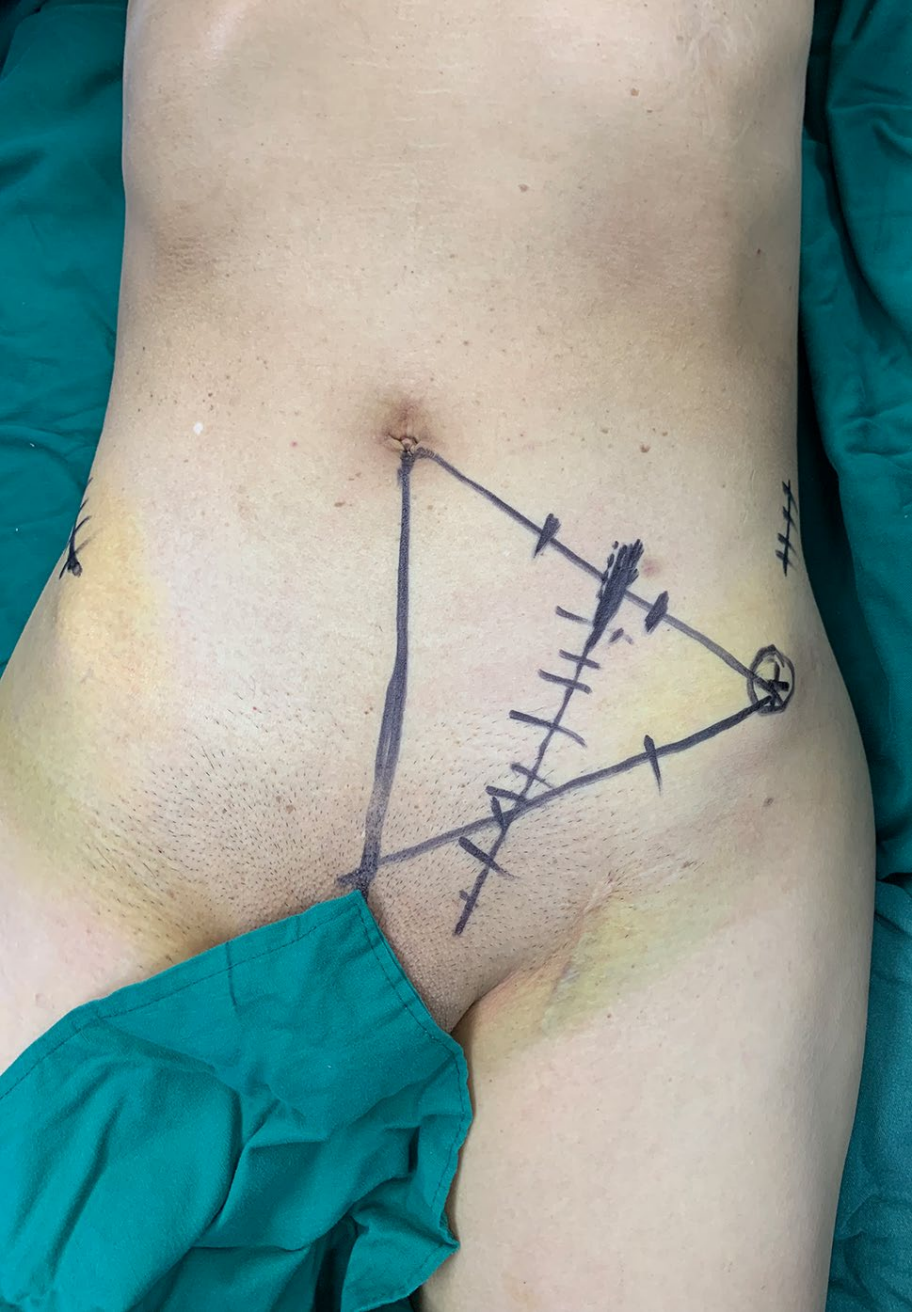
Surgical approach mark of pararectus approach group

In the transabdominal pararectus approach group, as shown in Fig. 2, the incision starts point were at the medial 2/3 and lateral 1/3 on the line joining the umbilicus and the anterior superior iliac spine, and the arc goes down to the medial 1/3 of the connecting line between the anterior superior iliac spine and the symphysis pubic. The incisions were made through the skin and subcutaneous adipose tissue and then the aponeurosis of obliquus externus abdominis were separated, followed by the exposure of the anterior sheath of musculus rectus abdominis, abdominal external oblique muscle, and semilunar line. The spermatic cord or uterine soft ligament was separated and carefully protected. Then, the rectus abdominis space was bluntly separated and the injury of the inferior epigastric artery were prevented. The internal oblique muscle and transverse abdominis muscle were lifting and the extraperitoneal space were exposed. After that, the iliac fossa, iliopubic eminence, pubic symphysis, quadrilateral plate, and sacroiliac joint were exposed successively through the first, second and third operation windows. Specifically, the exposure of the iliac fossa below iliac spine was the first window; On the surface of iliopsoas muscle, the exposure of the pelvic margin through the iliopubic eminence was the second window; Blunt separation of external iliac vessels, femoral nerve, and iliopsoas, the exposure of the superior ramus of pubis and pubic symphysis was the third window. The iliopsoas muscle, external iliac vessels, and femoral nerve were protected in the field of vision of the second and third windows. After exposure of the greater sciatic foramen and sacroiliac joint, the fractures were reduced by large-size reduction forceps or bucking bar and fixed by Kirschner wire temporarily, followed with fixed with locking plate or cannulated screw. Through the third window, the corona mortis can be seen directly and should be ligated if necessary, Besides, the posterior column of the acetabulum can be fixed by plate-screw. During the operation, attention should be paid to the protection of external iliac vessels and femoral nerves. The effects of fracture reduction and internal fixation were further confirmed by DR. After washing the wound repeatedly and stopping bleeding completely, a negative pressure drainage tube was allowed to be left at the operation site, externally connecting with a negative pressure drainage device, followed with stitched the incision carefully.

In the modified Stoppa approach group, a transverse incision was made on the fracture side above the perineum, or a longitudinal incision with a length of approximately 10 cm was made in the middle of the lower abdomen, the skin and subcutaneous soft tissue were cut. The abdominal white line was cut longitudinally, and an assistant pulled the incision to both sides. Subsequently, the peritoneum was pushed upward to expose and stretch the inferior abdominal wall muscle, iliopsoas muscle, femoral nerve, and external iliac vessels to expose the true pelvic margin. After the fracture was fixed with a steel plate or cannulated screw, intraoperative DR fluoroscopy was used to confirm the effect of reduction and internal fixation. After confirming the ideal position, the wounds were repeatedly rinsed, the bleeding stopped, indwelling drainage tube placed, and the incision sutured and closed. The operation was then completed.

After surgery, anticoagulant and analgesic drugs were used routinely. Antibiotics were used for 2–4 days, drainage was maintained for 48 h. Passive exercise of the lower limb joint and functional exercise of the quadriceps femoris was started on the first day after the operation, gradually transferring to active exercise and hip joint mobilization training. Crutches were used for 6–8 weeks or more postoperatively, and full weight-bearing was achieved 3 months after the operation.

### Clinical and Radiological Assessment

**Fig. 3:**
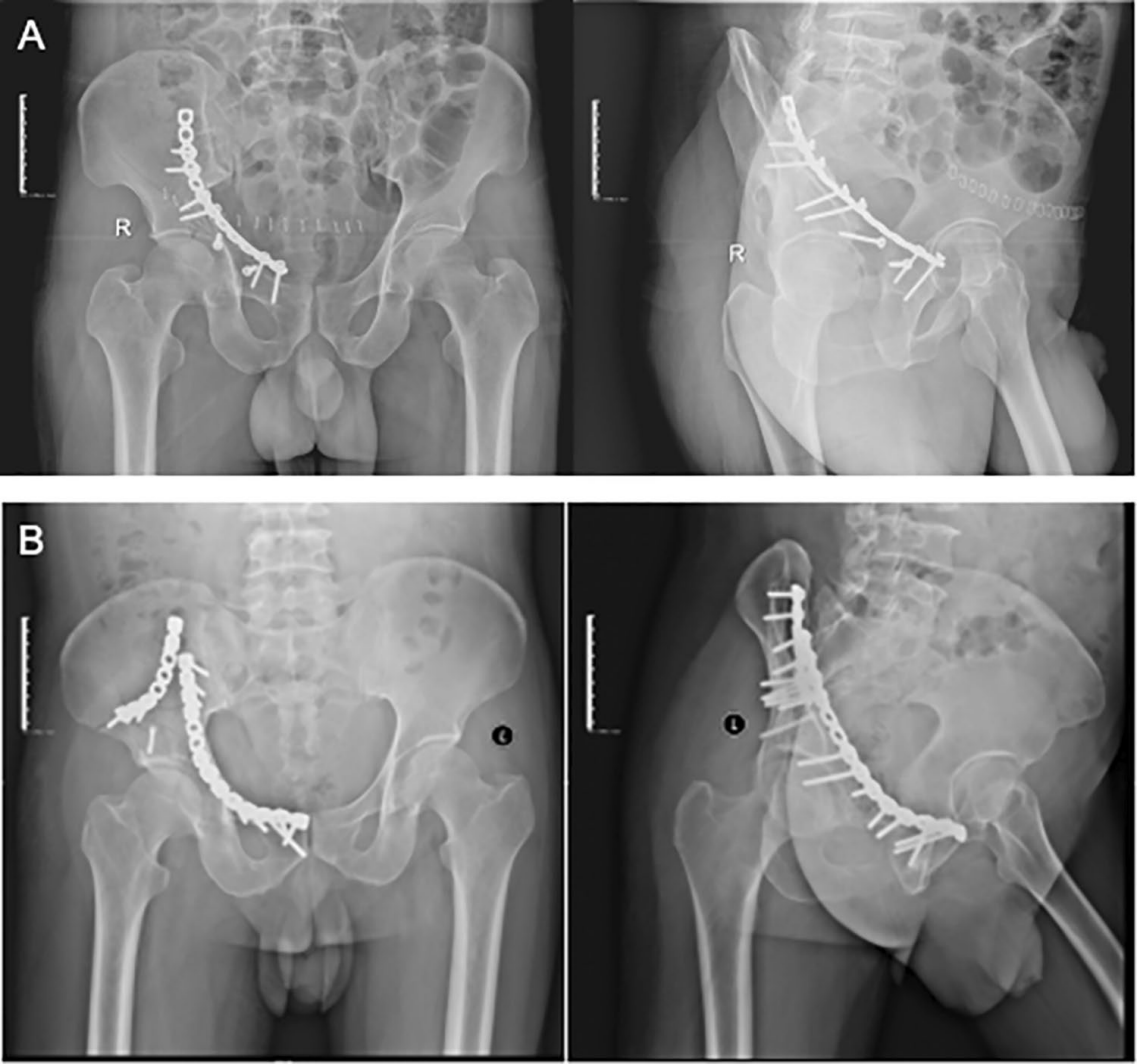
X-ray of the typical patient in **A** Stoppa approach group and **B** pararectus approach group

The operation time, incision length, blood loss (including blood loss during operation and amount of post-operation drainage), hospitalization time, and postoperative complications were recorded. According to the postoperative DR examination (Fig. 3), acetabular fracture displacement less than 2 mm and fracture piece separation distance less than 4 mm were judged as excellent; acetabular fracture displacement of 2–3 mm, and maximum separation distance of 4–10 mm were judged as good; The displacement of acetabular fracture was 2–3 mm, the maximum separation distance was 11–20 mm were judged as fair; the acetabular fracture displacement was more than 3 mm, and the maximum separation distance was more than 20 mm were judged as poor. The calculation formula of fracture reduction satisfaction is as follows: Fracture reduction satisfaction = (excellent cases + good cases)/total cases × 100%. Besides, according to Merled'Aubigné-Postel hip score, the recovery of hip function was evaluated 6 months after operation [13, 14].

### Statistical Analysis

The SPSS 11.0 software was used for statistical analysis, and mean value was used for measurement data ± standard deviation (x ± s). Two independent sample *t* tests were used to compare the two groups, and the chi-squared test was used to compare the count data. The difference was statistically significant at *p* < 0.05.

## Results

The operation time, incision length, blood loss, and average hospital stay were greater in the modified Stoppa approach group than in the pararectus approach group (Table 2). The difference between the two groups was statistically significant (*p* < 0.05).

**Table 2:** Comparison of therapeutic indexes between pararectus approach and Stoppa approach

Group	Case	Operation time /min	Incision length /cm	Operative blood loss /mL	Fracture reduction satisfaction (*n*, %)	Excellent and good rate of hip function score (*n*, %)	Incidence of complications (*n*, %)
Pararectus abdominis approach	30	180.000 ± 41.105	8.667 ± 1.373	259.667 ± 125.382	28 (93.33)	27 (90.00)	1 (3.33)
Stoppa approach	30	202.667 ± 32.793	11.600 ± 1.958	353.667 ± 156.590	25 (83.33)	25 (83.33)	3 (10.00)
Statistical test quantity		*t* = − 2.361	*t* = − 6.718	*t* = − 2.567	x^2^ = 1.456	x^2^ = 0.577	x^2^ = 1.071
*P* value		0.022	0.000	0.013	0.424	0.706	0.612

Both groups were followed up for 6 to 7 months, with an average of around 6.5 months. According to the above-mentioned standards of fracture reduction satisfaction, the degree of fracture reduction satisfaction in the pararectus approach group was 28/30. In the Stoppa approach group, the degree of fracture reduction satisfaction was 25/30. The excellent and good rate of hip function score in the pararectus approach group was 27/30, and that of the Stoppa group was 25/30. Hence this score was better in the pararectus approach group than in the Stoppa approach group, however, this difference was not significant (*p* > 0.05). In addition, there was no significant difference (*p* > 0.05) in satisfaction after fracture reduction between the two groups (Table 2).

In the pararectus approach group, there was a case of urinary tract infection, while in the Stoppa approach group, there was a case each of deep vein thrombosis, urinary tract infection, and postoperative incision superficial infection. There were no injuries to the sciatic, femoral, and lateral femoral cutaneous nerves, plate and screw loosening, fracture displacement, or heterotopic ossification in either group. Though the incidence of complications and fracture reduction unsatisfaction in the pararectus approach group was lesser than that in the Stoppa approach group, but the results have no statistical significance (*p* > 0.05). The incision length, operation time, and blood loss in the pararectus approach group were better compared to those in the Stoppa approach group, and the difference was statistically significant (*p* < 0.05).

## Discussion

In our clinical practice, we found that through the pararectus approach, using a single incision in the extraperitoneal space can expose the interior of the pelvis. This approach does not require dissection of the inguinal canal, the surgical trauma is minimal, and the surgical incision is small. It can fully expose the main blood vessels and nerves with less soft tissue dissection and adequately expose the fracture during the operation. Similarly, it does not need repeated changes of the retractor’s position or changes to the operation window. It can effectively avoid injury caused by intraoperative traction and reduce the possibility of major vascular and nerve injury [15]. Through this approach, we can observe whether there are corona mortis (the communicating branch between the external iliac vessels and obturator vessels) under direct vision. We can safely separate, cut, and ligate the corona mortis before dealing with the quadrilateral plate to avoid the difficulty of hemostasis caused by accidental cutting or tearing of the corona mortis. In addition, the pararectus abdominis approach can expose the entire true pelvic ring in the operation field, which greatly facilitates the cross quadrilateral plate or the arched line area shaping and the installation of the pelvic reconstruction plate. There is also a more reliable fixation of the acetabular quadrilateral plate, with free control of the direction of the electric drill and easy placement of double screw forceps, making it conducive for accurate placement of the plate and screw. When the quadrilateral plate fracture is exposed through the pararectus approach, the direction of the approach is perpendicular to the displacement of the fracture. This helps in better application of the reduction force to the ischial margin and thereby achieve the best reduction effect in the treatment of quadrilateral plate fracture rotation displacement. The results of this study also showed that the operation time, incision length, blood loss, and hip function score of the pararectus approach group were significantly better than those of the modified Stoppa approach, which further confirmed the important application value of this approach in the surgical treatment of complex pelvic acetabular fractures [14, 16, 17].

Compared with the traditional ilioinguinal approach and the modified Stoppa approach, the transabdominal pararectus approach has many advantages, such as less trauma, less involvement of important nerves and blood vessels, and less tissue damage. At the same time, transabdominal pararectus approach is convenient for fracture reduction and fixation, owing to the incision is located on the same side of the fracture, which is close to the acetabulum and can be directly observed at the front of the acetabulum [18, 19]. Keel et al. reported that in the treatment of 48 cases of acetabular fractures by pararectus approach, and they found that this approach provided clear visualization of the fracture site and reduced the damage of skin, muscle, and other soft tissues with an average incision length of 11 cm [20]. Bastian et al. found that the pararectus approach can expose more pelvic structures and complete the fixation of posterior ring of pelvis fractures without additional auxiliary approaches [21]. Besides, during the operation, the incision near the rectus abdominis are convenient for fracture reduction and fixation. Trans-verse and T-shaped fractures involving the posterior column can be fixed with long screws through the posterior column, so as to achieve simultaneous fixation of the anterior and posterior column through only one incision, while it is difficult to implant and fix the posterior column screw through the Stoppa approach.

However, the pararectus approach also has some limitations. First, for pelvic acetabular fractures combined with posterior acetabular wall fractures, pararectus approach often need to be coordinated with Kocher-Langenbeck approach, which may result in additional surgical trauma and increases the operation time. Secondly, the pararectus approach may cause the damage to the rectus abdominis innervation, and thus may lead to poor wound healing, muscle atrophy, and even ventral hernias [22]. Thirdly, in case of severe extraperitoneal adhesion or difficult exposure of the pararectus approach, the ilioinguinal approach should be used instead [23]. Besides, this approach has the risk of destroying the peritoneum, which is mostly related to the operators were unfamiliar with the abdominal anatomical structure. Hence, the operator must be familiar with the various anatomical layers of the abdominal cavity, and should suture peritoneum promptly once find the operation mistakes of peritoneal rupture. Lastly, this approach is not suitable for patients with severe osteoporosis, because the patients may have early loosening of internal fixation or refracture during reduction and fixation [24, 25].

In summary, the pararectus approach can reveal better the anatomical structure of the pelvis and acetabulum, such as the corona mortis and quadrilateral plate, for conducive fracture reduction and fixation. It can also effectively shorten the length of the incision, reduce intraoperative blood loss, and shorten the operation time. It is a good choice for the treatment of complex pelvic acetabular fractures [26]. Besides, with the rise of digital orthopedic technology, researchers and clinical staff globally are using 3D printing technology in the surgical treatment of complex pelvic and acetabular fractures. If combined with the pararectus approach, it will further reduce the operation time and injury, improve the quality of reduction, and improve the surgical effect, which may be a new direction for the surgical treatment of complex pelvis and acetabulum fractures in the future [27]. Lastly, the actual effects of this approach for the treatment of pelvic and acetabular fractures still need further clinical verification, because the pararectus approach has not been used for a long time in clinical practice, the number of reported cases is limited, and there is a lack of long-term postoperative follow-up. However, we believe that the pararectus approach can provide a new choice for the clinical treatment of acetabular fractures.

## Conclusions

We retrospectively analyzed the outcomes of 60 cases of pelvic acetabular fractures treated by pararectus approach or modified Stoppa approach. And the results showed that the operation time, incision length, and blood loss of the pararectus approach group were significantly better than those of the modified Stoppa approach. Besides, the pararectus approach can reveal the better anatomical structure of the pelvis and acetabulum, such as the corona mortis and quadrilateral plate, for conducive fracture reduction and fixation. So all these results furtherly confirmed the important application values of the pararectus approach in the surgical treatment of complex pelvic acetabular fractures. It is expected that this approach will be more widely used in the clinical treatment of pelvic and acetabular fractures in the future.
